# A novel electron paramagnetic resonance-based assay for prostaglandin H synthase-1 activity

**DOI:** 10.1186/1476-9255-3-12

**Published:** 2006-09-28

**Authors:** Catriona M Turnbull, Danny McClure, Adriano G Rossi, Ian L Megson

**Affiliations:** 1 Centre for Cardiovascular Science, Queen's Medical Research Institute, University of Edinburgh, Edinburgh, UK; 2 MRC Centre for Inflammation Research, Queen's Medical Research Institute, University of Edinburgh, Edinburgh, UK; 3 Free Radical Research Facility, UHI Millennium Institute, Inverness, UK

## Abstract

**Background:**

Prostaglandin H_2 _synthase (PGHS) is the enzyme that catalyses the two-stage conversion of arachidonic acid to prostaglandin H_2 _(PGH_2_) prior to formation of prostanoids that are important in inflammation. PGHS isozymes (-1 and -2) are the target for nonsteroidal anti-inflammatory drugs (NSAIDs).

Given the rekindled interest in specific anti-inflammatory PGHS inhibitors with reduced unwanted side effects, it is of paramount importance that there are reliable and efficient techniques to test new inhibitors. Here, we describe a novel *in vitro *electron paramagnetic resonance (EPR)-based assay for measuring the activity of PGHS-1.

**Methods:**

We validated a novel *in vitro *PGHS-1 activity assay based on the oxidation of spin-trap agent, 1-hydroxy-3-carboxy-pyrrolidine (CPH) to 3-carboxy-proxy (CP) under the action of the peroxidase element of PGHS-1. This quantifiable spin-adduct, CP, yields a characteristic 3-line electron paramagnetic (EPR) spectrum.

**Results:**

The assay is simple, reproducible and facilitates rapid screening of inhibitors of PGHS-1. Aspirin (100 μM, 1 mM) caused significant inhibition of spin-adduct formation (72 ± 11 and 100 ± 16% inhibition of control respectively; P < 0.05). Indomethacin (100 μM) also abolished the signal (114 ± 10% inhibition of control; P < 0.01). SA and the PGHS-2-selective inhibitor, NS398, failed to significantly inhibit spin-adduct generation (P > 0.05).

**Conclusion:**

We have demonstrated and validated a simple, reproducible, quick and specific assay for detecting PGHS-1 activity and inhibition. The EPR-based assay described represents a novel approach to measuring PGHS activity and provides a viable and competitive alternative to existing assays.

## Background

**Figure 1:**
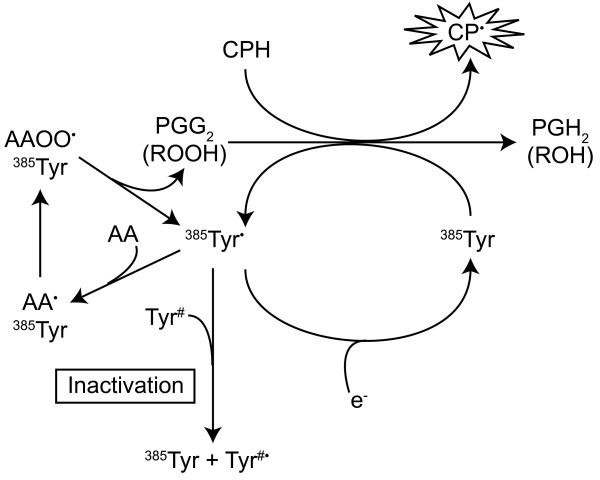
Schematic diagram showing the peroxidase activity of PGHS. The process requires prior formation of a tyrosine radical from a tyrosine residue in close proximity to the haem group (Tyr 385). The tyrosyl radical is either recycled or participates in the suicide inactivation of the enzyme (for review of this process see [39]. Following incorporation of oxygen and formation of PGG_2_, the peroxidase reduces the peroxyl moiety to the equivalent alcohol. The process allows for the concomitant oxidation of spin-trap CPH to CP which is detected by EPR.

Prostaglandins are derived from arachidonic acid (AA) in a pathway dependent on the PGHS (EC 1.14.99.1) family of enzymes, which are commonly known as cyclooxygenase (COX), referring to the first step of enzymatic activity. PGHS converts AA to prostaglandin H_2 _(PGH_2_), the precursor of all prostanoids. The enzyme contains two active sites: a COX site, where AA is converted into the hydroperoxy endoperoxide, prostaglandin G_2 _(PGG_2_), and a haem with peroxidase activity that reduces PGG_2 _to PGH_2 _(For review see [1]). The reduction of PGG_2 _by the peroxidase element generates the corresponding alcohol. This reaction has previously been demonstrated to concurrently oxidise aminopyrine molecules to aminopyrine free radicals [2]. Here, a spin-trapping agent, 1-hydroxy-3-carboxy-pyrrolidine (CPH) is oxidised to 3-carboxy-proxy (CP), probably under the action of the peroxidase, in a similar fashion to that previously seen with aminopyrine (Fig. 1).

Two structurally similar PGHS isoforms exist (PGHS-1 and PGHS-2) which are encoded by different genes and the expression of which varies between tissues [3]. PGHS-1 is often referred to as the 'house-keeping' isoform due to its regulatory functions in many tissues. PGHS-2 is virtually undetectable under normal conditions in most tissues and is often referred to as the 'inducible' isoform due to its tendency to be expressed in response to inflammatory stimuli. The exception to this is in the brain and spinal cord, where PGHS-2 is constitutively expressed and plays a role in nociception signaling [4].

The importance of PGHS as a therapeutic target has long been highlighted by the actions of aspirin, [5,6] the first drug of the family of nonsteroidal anti-inflammatory drugs (NSAIDs) for use as analgesics, anti-inflammatory agents and antithrombotic agents. In contrast to other NSAIDs, such as indomethacin, which reversibly bind at the COX active site [7], aspirin causes an irreversible inhibition of PGHS by rapidly and selectively acetylating the hydroxyl group of a serine residue (Ser 530) near the C-terminus of the enzyme, forming an impediment to the binding of AA [8-10]. The ensuing irreversible PGHS inhibition requires *de novo *synthesis of the enzyme for subsequent production of prostaglandins.

Interest in PGHS has been re-ignited recently on account of two advances in the development of novel NSAIDs. Firstly, nitroaspirins [11-14] are being developed in an effort to overcome the gastrotoxic side-effects of aspirin that represent the major limitation to its therapeutic use [15-17]. Nitroaspirins make use of the protective effects of nitric oxide (NO) to compensate for the potentially damaging impact of aspirin-mediated depletion of protective prostaglandins in the gastric mucosa. Secondly, the suggestion that the gastrotoxic side effects of aspirin are due to the inhibition of housekeeping PGHS-1, whereas its anti-inflammatory effects are due to inhibition of PGHS-2, led to the development of selective inhibitors of the COX-2 activity of PGHS-2, in the hope that the beneficial effects could be retained without injury to the gastric mucosa [18]. However, recently, several of these new selective PGHS-2 inhibitors have been withdrawn due to mounting evidence of an increased risk of stroke. The increased risk of thrombus is thought to be due to inhibition of PGHS-2 in endothelial cells leading to down regulation of anti-thrombotic prostaglandins (such as prostacyclin) in relation to the unaffected PGHS-1 derived thromboxanes [19-21].

Given the intense interest in PGHS activity and inhibition, it is perhaps surprising that relatively few assays for the activity of this enzyme have been developed. Amongst the most popular techniques is the measurement of thromboxane B_2 _(TXB_2_), the stable metabolite of PGH_2_-derived TXA_2_, as a marker of PGHS activity [22]. Alternatively, measurement of oxygen consumption using an oxygen electrode [23,24], assays using radio-labelled substrate [25] and immunoassays for the prostaglandin products [23] can also be applied. More recently, a commercial chemiluminescent assay (Axxora, Nottingham, UK) has been developed for use on purified enzyme. The assay involves the use of labelled substrate that generates a luminescent product under the action of the hydroperoxidase element of PGHS, most likely via the generation of oxidising free radicals.

Electron paramagnetic resonance (EPR) has been previously utilized to measure free radicals generated by PGHS as a measure of its activity [26,27]. However these techniques can involve complex mechanisms to trap the short-lived radical species. For instance in an *in vitro *assay using purified PGHS, liquid nitrogen was utilised to stop the reaction and was required to stabilise the tyrosyl radical species generated, in order that it could be recorded by EPR [26]. Another technique which recorded PGHS activity in mouse keratinocytes relied on the use of the antioxidant glutathione to stabilize the generated radical before trapping it with DMPO [27].

Here, we present a novel method for assaying the activity of PGHS-1 in which EPR is used to detect the stable adduct CP, formed from oxidation of commonly available spin-trap, CPH [28], under the action of the peroxidase element of PGHS-1. The aim of the studies described was to validate this technique for assaying PGHS-1 activity *in vitro*, to determine its effectiveness at establishing the inhibitory effects of conventional NSAIDs and to confirm that a COX-2-selective inhibitor was ineffective in this assay.

## Methods

Unless otherwise stated, all drugs and chemicals were purchased from Sigma, Dorset, UK. The assay was performed at 37°C in 1 ml of Tyrode's buffer (137 mM NaCl, 2.7 mM KCl, 1.05 mM MgSO_4_, 0.4 mM NaH_2_PO_4_, 12.5 mM NaHCO_3_, 5.6 mM Glucose, 10 mM HEPES and 0.8 mM CaCl_2 _in deionised water at pH 7.4. 100 units/ml ovine seminal PGHS-1 was incubated with 1.5 μM haematin (5 min, 37°C) prior to the assay. Data from the manufacturers of the PGHS-1 reveals that 1 unit of enzyme consumes 1 nanomole of oxygen at 37°C in the presence of 1 μM haematin and 100 μM AA. Aspirin, salicylic acid, indomethacin, NS398 (Merck Biosciences, Nottingham, UK) or vehicle control (DMSO, 1%) was added and left to incubate for a further 10 min prior to addition of the spin-trap, CPH (1 mM; Axxora). At this point (t = -2 min), a baseline EPR measurement was taken (MS200, Magnettech, Germany. Instrument settings: B0-field, 3356 gauss; sweep width; 50 Gauss, sweep time, 30 sec; modulation amplitude, 1500 mGauss; microwave power, 20 mW). 2 min later, 100 μM AA (as sodium salt) was added (t = 0). Further EPR readings were taken at t = 1.5 (the earliest timepoint at which readings could consistently be taken after addition of AA), 4 and 6 min. The suicidal nature of PGHS-1 activation means that the period of activation is anticipated to be complete within ~1 min of AA addition [3].

The results are corrected for any auto-oxidation of spin-trap by subtraction of values recorded from a duplicate sample run in the absence of AA. The intensity scale on the y-axis of all graphs is an arbitrary scale based upon the area under the curve of the first derivative traces generated.

### Statistical analyses

All statistical tests were performed using GraphPad Prism version 4. P < 0.05 was considered to be statistically significant. Tests performed were either 1-way ANOVA with Dunnett's post-test or 2-way ANOVA, as indicated in the text.

## Results

### Time-dependent adduct generation by PGHS-1

**Figure 2:**
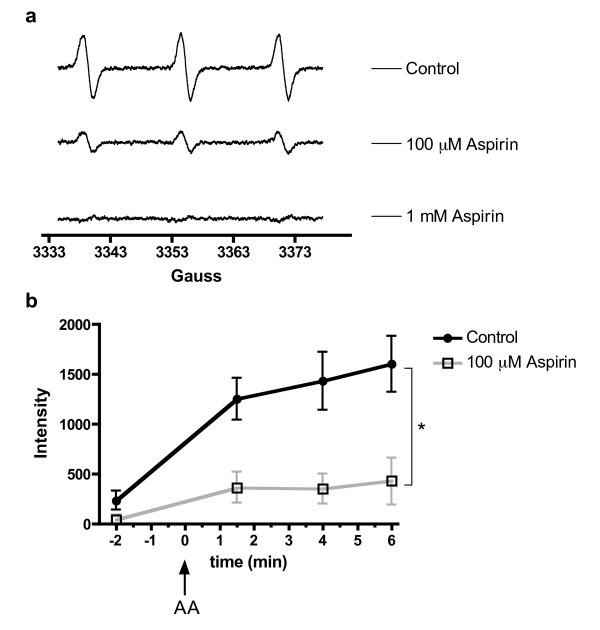
(a) Sample EPR spectra obtained in the absence (control; PGHS + AA) and presence of aspirin (100 μM or 1 mM) after correction for background autoxidation. EPR settings: B0-field, 3356 gauss; sweep width; 50 Gauss, sweep time, 30 sec; modulation amplitude, 1500 mGauss; microwave power, 20 mW. (b) Mean data for development of EPR signal intensity (AU) with time in the absence (control; PGHS + AA) and presence of aspirin (100 μM). In both cases, substrate (AA) was added at t = 0 min. P = 0.02, 2-way ANOVA, repeated measures: n = 9–12.

Addition of AA caused a time-dependent increase in the characteristic 3-line EPR spectrum for a spin-adduct with the unpaired electron in the vicinity of a nitrogen atom (Fig 2a). The majority of the reaction was complete by the time the first reading was taken (t = 1.5 min). The signal developed rapidly before the first reading (244 intensity units.min^-1^), but subsequently slowed to a relatively constant rate (65 intensity units.min^-1^) over the following 4.5 min of the assay (Fig 2b, n = 9–12); the equivalent experiment without AA failed to show the initial rapid rise and was significantly lower than the AA-treated sample throughout (p = 0.02, 2-way ANOVA, repeated measures). An inter-sample coefficient of variation of 0.18 (18%) was calculated from the control data obtained. From these data, it was determined that t = 1.5 min was an appropriate point at which to compare free radical generation between control and NSAID-treated PGHS-1, given that spin-adduct generation in response to AA had peaked – subsequent adduct formation was at an equivalent rate in control and AA-treated samples and was likely to be due to non-specific auto-oxidation of CPH.

### Inhibitory effect of aspirin and salicylic acid

**Figure 3:**
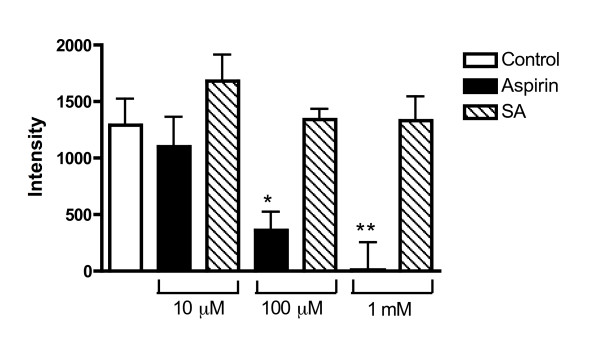
Effect of aspirin and salicylic acid (10 μM – 1 mM) on EPR signals generated from PGHS-1 after treatment with substrate (AA). In each case, incubations with aspirin or SA were for 10 min prior to the baseline EPR reading (t = -2 min, not shown). AA was added at t = 0 min and readings shown were taken at t = 1.5 min. *P < 0.05, **P < 0.01; 1-way ANOVA with Dunnett's post-hoc test vs. control: n = 8–10.

The impact of pre-incubation of PGHS-1 with different concentrations of aspirin that spanned the known therapeutic range (10 μM – 1 mM) is shown in Fig 3. Our results indicate that aspirin concentrations of 100 μM and 1 mM caused significant inhibition of spin-adduct formation at the 1.5 min time-point (to 72 ± 11 and 100 ± 16% of control respectively; P < 0.05 for both compared to control, 1-way ANOVA followed by Dunnet's post-hoc analysis).

Parallel experiments with salicylic acid (SA, 10 μM – 1 mM; n = 6) showed that a 10 min pre-incubation with SA failed to significantly inhibit generation of the spin-adduct, even at the highest concentration (P > 0.05).

### Comparative pharmacology of PGHS-1 and PGHS-2 inhibitors

**Figure 4:**
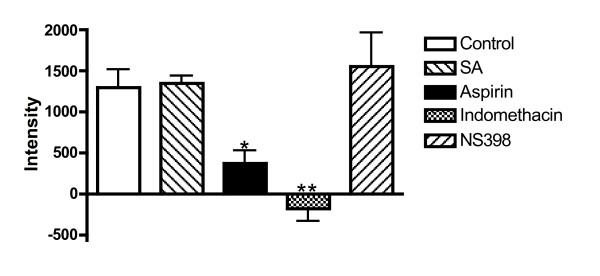
Comparative effects of SA and recognized NSAIDs (all 100 μM) on EPR signal intensity measured at t = 1.5 min. *P < 0.05, **P < 0.01; 1-way ANOVA with Dunnett's post-hoc test vs. control: n = 6–10.

Equivalent concentrations (100 μM) of the recognized non-selective PGHS inhibitors, aspirin and indomethacin both significantly (P < 0.05 and P < 0.01 respectively) inhibited generation of the EPR-detectable spin adduct at 1.5 min (72 ± 11 and 114 ± 10% inhibition of control response respectively), but the PGHS-2-selective inhibitor, NS398 had no effect (P > 0.05) on this assay of PGHS-1, despite its use at a concentration which is in excess of that required to significantly inhibit PGHS-2 [29] (Fig 4).

## Discussion

Here we have validated a new, quick, simple and reproducible method for detecting PGHS-1 activity and inhibition *in vitro*. The principle of the assay was based on the concomitant oxidation which occurs with the reduction of PGG_2 _by the peroxidase element of PGHS [2]. This oxidation can be exploited in the absence of antioxidant glutathione to oxidise spin-trapping agent CPH to the stable adduct, CP which generates a characteristic 3-line EPR spectrum. The amplitude of the EPR signal is proportional to the amount of adduct generated.

Our results indicated that oxidation of CPH by the isolated PGHS-1 enzyme upon addition of the enzyme substrate, AA, was sufficient to be easily detectable by EPR. The initial peak in the detected signal recorded at the first reading, had by the subsequent time-points slowed to a rate that was equivalent to the signal generation from substrate-free enzyme, most likely due to autoxidation of the spin trap. The loss of specific enzyme-mediated radical generation is unsurprising, given the well-recognised suicidal nature of activated PGHS-1 [3]. From these time-course experiments, we selected a 1.5 min time-point for subsequent comparative studies because by this time the AA-dependent free radical generation was complete but the signal was not significantly enhanced by autoxidation of the spin trap.

Aspirin pretreatment (10 min) was shown to have a concentration-dependent inhibitory effect in the published range [30], but the acetyl-free counterpart, SA, failed to significantly inhibit enzyme activity. The lack of effect of SA indicates that aspirin-mediated inhibition of the enzyme is dependent on the acetyl group, the moiety involved in PGHS inhibition, and not due to a non-specific antioxidant effect. SA was not expected to have any effect in this assay because it is known not to affect PGHS-1 or 2 activity [31] except in intact cells [30], possibly by suppressing PGHS-2 transcription in response to exogenous stimuli [32]. Furthermore, the reversible, non-specific PGHS inhibitor, indomethacin [33] was demonstrated to have a powerful inhibitory effect, whilst the PGHS-2 specific inhibitor, NS398 at a concentration known to inhibit PGHS-2 [29] failed to do so. Signal intensities shown in the figures are after subtraction of an AA-free control from a parallel experiment to account for autooxidation of the spin trap. A negative signal (as observed with indomethacin for example) therefore indicates that the control value was greater than the AA-treated sample, which might suggest that AA has a slight antioxidant effect on its own.

These results confirm that the assay is relevant for NSAIDs with different modes of inhibitory action. As purified PGHS-2 is now available commercially (Sigma), it may be possible to adapt this assay system to help determine the specificity of novel PGHS-2 inhibitors.

This assay provides a convenient screening method for inhibitors of the PGHS enzyme. Whilst various techniques exist to assay the activity of PGHS isoforms, each has its own disadvantages. For instance, recording the oxygen uptake by PGHS is an option to measure its activity but this requires high enzyme concentrations and also accurate control of the initial oxygen concentrations [24]. Optical techniques are prone to interference from coloured assay constituents (such as haematin) and require high AA concentrations. Techniques recording uptake of radiolabelled substrate [25] can be complex and expensive. Our technique provides quick inexpensive results that give real-time determination of COX-inhibition. Data obtained, as demonstrated by percentage inhibition of control response achieved with aspirin, is comparable to other *in vitro *techniques [34-36].

The technique described in the present study also offers a simpler alternative than previous EPR techniques where generated radicals are detected following complex radical stabilization steps [26,27] and thereby providing potential for less loss of signal. Tsai *et al*. [26] used dry ice and liquid nitrogen to stop the reaction and trap the radical. The recording of spectra was then carried out under liquid nitrogen. By comparison, our method uses direct oxidation of a spin-trap to generate an adduct that is sufficiently stable to allow successive time-point readings to be taken without the need to freeze the sample at the required time-point. The method by Schreiber *et al*. [27] did use a spin-trap, but required the use of glutathione to reduce an amine radical to the parent amine, liberating a thiyl radical which was trapped by DMPO. In our method, the spin-trap is oxidised directly, thus reducing the potential for loss of signal. Furthermore, there is some evidence of rapid signal decay in the Schreiber method; the spectra show a decrease of the DMPO-thiyl signal during the course of the measurement, whereby the spectral lines no longer conform to the expected 1:2:2:1 ratio. By comparison, the EPR signal generated in the present technique is much more resilient and does not decay during the time-course of experiments.

It is important to recognize the potential limitations of this assay. Its reliance on the oxidation of CPH might preclude its use in cells, given that the cellular environment is usually very rich in antioxidants such as GSH, and its presence at intracellular concentrations (~5 mM) could effectively compete out the spin-trap and nullify the assay. The susceptibility of polyunsaturated fatty acids such as AA to peroxidative attack by reactive oxygen species may impact on the recorded level of radical if radicals are consumed by free AA in the samples [37,38]. Furthermore, it is important to recognize that the *in vitro *nature of the assay does not account for any absorption, metabolic or availability issues that might relate to applied NSAIDs.

## Conclusion

In summary, we have validated a new, simple, EPR-based assay for detecting PGHS-1 activity and inhibition. We have demonstrated it to be sensitive to the inhibitory effects of conventional NSAIDs (aspirin and indomethacin) and not to SA or a PGHS-2 specific inhibitor. In principle, this assay should be equally applicable to measuring PGHS-2 activity in isolated enzyme. As such, this assay might prove to be a useful research tool in the ongoing search for novel PGHS inhibitors of both isoforms of the enzyme.

## Abbreviations

**AA**, arachidonic acid; **COX**, cyclooxygenase; **CPH**, 1-hydroxy-3-carboxy-pyrrolidine; **EPR**, electron paramagnetic resonance; **NO**, nitric oxide; **NSAID**, nonsteroidal anti-inflammatory drugs; **PGG_2_**, prostaglandin G_2_; **PGH_2_**, prostaglandin H_2_; **SA**, salicylic acid; **TXB_2_**, thromboxane B_2_.

## Competing interests

The author(s) declare that they have no competing interests.

## Authors' contributions

Experimental design and procedures were performed by CMT and assisted by DM. AGR and ILM supervised experimental design and procedures. AGR and ILM oversaw manuscript construction, revising it critically for important intellectual content. All authors have given final approval of the version to be published.
